# Supplementary material for: Stxbp1/Munc18-1 haploinsufficiency impairs inhibition and mediates key neurological features of STXBP1 encephalopathy
Source: eLife. 2020 Feb 19;9:e48705. doi: 10.7554/eLife.48705 (PMC7056272; doi:10.7554/eLife.48705)
Supplement: Supplementary file 2. — The phenotyping tests in mouse models (the second column) are grouped based on the clinical features of STXBP1 encephalopathy (the first column). The results of phenotyping tests from different mouse models and studies are presented in the table. [file elife-48705-supp2.docx]

**Table S2. Phenotypic comparison of *STXBP1* encephalopathy patients and mouse *Stxbp1* haploinsufficiency models**

| Human patient phenotypes  (% of patients^1^) | Mouse phenotyping tests | Mouse *Stxbp1* haploinsufficiency models and phenotypes^2^ | | | | | | |
| --- | --- | --- | --- | --- | --- | --- | --- | --- |
|  |  | Deletion of exon 7: *tm1d* (C57BL/6J) | STOP after exon 6: *tm1a* (C57BL/6J) | Deletion of exon 3 (C57BL/6N) | Deletion of exons 2–6 (129Sv) | Deletion of exons 2–6 (mix of 129Sv and C57BL/6J) | Deletion of exons 2–6 (C57BL/6J) | Deletion of exon 2 (C57BL/6J) |
|  |  | This paper | This paper | Miyamoto et al., 2017 | Orock et al., 2018 | Kovačević et al., 2018 | | |
| – | Stxbp1 protein reduction in brain tissues  (Western blot) | 40–50% in cortex, hippocampus, thalamus + hypothalamus, striatum, midbrain + hindbrain;  30% in cerebellum;  20% in olfactory bulb | 40–50% in cortex, thalamus + hypothalamus, striatum;  30% in hippocampus, midbrain + hindbrain, cerebellum;  20% in olfactory bulb | 25% in cortex,  50% in hippocampus | 25% in brain | – | – | – |
| Epilepsy (95%) | Video-EEG/EMG | Yes | – | – | – | – | Yes | Yes |
| Intellectual disability (100%) | Novel object recognition | Yes | Yes | – | – | – | – | – |
|  | Contextual fear | Yes | Yes | – | – | – | – | – |
|  | Cued fear | Yes | Yes | Yes | – | – | – | – |
|  | Intellicage | – | – | – | Yes | – | – | – |
|  | Radial arm water maze | – | – | – | Yes | – | – | – |
|  | Barnes maze | – | – | – | – | – | Yes | No |
|  | Morris water maze | – | – | – | – | No | – | – |
| Motor deficits  (92%) | Hindlimb clasping | Yes | Yes | – | – | – | – | – |
|  | Grip strength | Yes | Yes | – | – | No | No | No |
|  | Foot slip | Yes | Yes | – | – | – | – | – |
|  | Vertical pole | Yes | Yes | – | – | – | – | – |
|  | Rotarod | No^3^ | – | – | – | – | No | No |
| Developmental delay (64.3%) | Body weight | Yes | Yes | – | – | Yes | Yes | Yes |
| Hyperactivity (4%) | Open-field | Yes | Yes | No | – | Yes | No | No |
| Autistic traits (17%) | Hole-board | Yes | Yes | – | – | – | – | – |
|  | Three-chamber | No | No | No | – | – | No | No |
|  | Partition | No | – | – | – | – | No | No |
| Aggressive behavior (3.4%) | Resident- intruder | Yes | Yes | Yes | – | – | – | – |
|  | Tube | Yes | Yes | – | – | – | – | – |
| Anxiety  (27%) | Elevated plus maze | Yes | Yes | No | – | – | Yes | Yes |
|  | Open-field | Yes | Yes | No | – | Yes | No | No |
|  | light-dark chamber | Yes | Yes | Yes | – | – | Yes | Yes |
| N/A | Nest building | Yes | Yes | – | – | – | – | – |
|  | Marble burying | Yes | Yes | – | – | – | – | – |

^1^Percentage is based on Stamberger et al., 2016 except for anxiety, which is based on Suri et al., 2017.

^2^For the phenotypes of mouse models, “Yes” indicates a statistical difference between WT and mutant mice, “No” indicates that no statistical differences were detected between WT and mutant mice, and “–” indicates that the test was not performed.

^3^Mutant mice performed better than WT mice at the age of 6–7 weeks.
